# Pater *Knows Best: Withdrawal of Medical Treatment from Infants in Scotland*

**DOI:** 10.1093/ojls/gqaa019

**Published:** 2020-09-03

**Authors:** Jonathan Brown, Sarah Christie

**Keywords:** parens patriae, infants, medical treatment, withdrawal, best interests, Scots law

## Abstract

The cases of Charlie Gard and Alfie Evans placed the withdrawal of treatment from terminally ill infants at the forefront of medical law and ethics. In the medico-legal context, Scottish court procedures materially differ from those in England. This article considers these differences in light of the possibility that a similar case might soon be called before the Scottish courts. The Court of Session would then be required to consider whether to utilise its *parens patriae* jurisdiction to consent to the withdrawal of treatment as if it were the parent of the infant. The operation of this jurisdiction is such that the outcome of any Scottish case cannot be said to be certain, as the Scottish courts are bound to pay more heed to parental autonomy than their English counterparts do.

## 1. Introduction

The cases of Charlie Gard^1^ and Alfie Evans^2^ placed the question of withdrawing treatment from terminally ill infants at the forefront of medical law and ethics.^3^ Though the law might have been ‘compassionately and correctly applied’ in these cases,^4^ this ostensible clarity did not prevent protracted litigation,^5^ nor an outpouring of strong public feeling.^6^ Both cases commanded significant media attention and attracted comment from public figures, politicians and even the Pontiff.^7^ The crux of many of the complaints concerning the legal process was the apparent sidelining of the parents’ wishes.^8^ Recognising this, it is submitted that the Scottish Court of Session would be faced with a greater legal and ethical conundrum—and potentially greater public furore—than the courts of England and Wales faced when applying the relevant law relating to the withdrawal of medical treatment.

The legal test to be applied in ‘futile’ circumstances^9^ asks whether the withdrawal of treatment would be in the child’s ‘best interests’.^10^ This metric is the same in Scotland as it is in the rest of the UK.^11^ Perhaps for this reason, academic commentary concerns itself with ‘British’ courts, without distinguishing between the legal systems of the UK.^12^ The conceptual framework underpinning the law on withdrawal of treatment rests on a fundamentally distinct basis in Scotland, however, since the Court of Session retains *parens patriae* (‘parent of the nation’)^13^ jurisdiction over Scottish legal subjects.^14^ It is not, therefore, accurate to say that physicians in Scotland have the power ‘to unilaterally withdraw or withhold treatment that they regard to be futile’;^15^ rather, physicians possess a *de facto* privilege, due to the flexible interpretation of the law following from *Law Hospital NHS Trust v Lord Advocate*,^16^ to do so within certain parameters.^17^ In practical terms, to suggest that this position differs greatly from that in England is (ordinarily) no more than hair-splitting,^18^ but in theoretical terms the gulf between Scots and English law is vast.^19^

The greatest consequence of Scotland’s continued recognition of the *parens patriae* jurisdiction is that, while in England and Wales the courts ‘can only issue a declarator as to the legality of a proposed course of conduct’,^20^ in Scotland the Court of Session can refuse treatment ‘on behalf of the incapax’.^21^ Ultimate parental authority, therefore, is vested not in the parents, but rather in the judiciary.^22^ As ‘the contentious aspect of the case[s of *Gard* and *Evans*] is the issue surrounding parental autonomy’,^23^ it follows that the Scottish courts are placed in an awkward position. While the courts in England and Wales may legitimately say that they do no more than declare the conduct of the clinicians lawful in cases concerning the withdrawal of medical treatment, to achieve the same ends in such cases the Scottish courts must go further and expressly rule that the views of any child’s parents are subordinate to those of the court. This paper does not propose to determine whether this is ethically justifiable; rather, it asks whether, due to the differences in the law, and conscious of considerations of public policy and public perceptions concerning the importance of parental authority and autonomy, the Court of Session might decide a case such as *Gard* or *Evans* differently.

## 2. Gard *and* Evans

### A. The Factual Background

Charlie Gard had been diagnosed with a rare disease^24^ which had rendered him severely disabled. His prognosis was described as dire and against this backdrop, his parents accepted that his life at present was not worth sustaining unless there was some available treatment. Contrary to medical advice, they wished to take him abroad for entirely experimental and untested therapy, which even the consultant in question viewed as extremely unlikely to trigger any improvement, and as only holding out a theoretical possibility of working. Alfie Evans was diagnosed with a progressive neurological condition and a similar prognosis to Charlie. His doctors had determined that there was no longer any viable treatment to improve his condition or halt his decline, and sought a declarator from the court that it was no longer lawful to continue ventilation, as it was not in his best interests. His parents wanted to take him abroad, to be able to continue life support for longer, followed ultimately by taking him home to die within their own timeframe.

Francis J heard the case of *Great Ormond Street Hospital v Yates and others*,^25^ and set out a clear statement about the court’s involvement in such cases:

Some people might ask why the court becomes involved at all, why should the parents not be the ones to decide? A child’s parents having parental responsibility have the power to give consent for their child to undergo treatment, but overriding control is vested in the court exercising its independent and objective judgment in the child’s best interests.^26^

In asserting that ‘best interests’ is the only test to be applied here, the judge used well-established case law^27^ to reinforce the need for the court to make an objective assessment of what is in the child’s best interests, balancing the competing factors and using a ‘balance sheet approach’ where appropriate.^28^ He also highlighted the dicta on best interests in *Aintree University Hospital NHS Foundation Trust v James*,^29^ on withholding or withdrawing treatment from an incompetent adult patient. There it was emphasised that, under section 1(5) of the Mental Capacity Act 2005, the assessment of best interests is based on whether it is in the patient’s best interests to be given the treatment, not whether it is in their best interests for it to be withdrawn. This relies on the nature of consent in these cases. The court consents on behalf of the incompetent patient, and if the treatment is not in that patient’s best interests, there can be no question of the court consenting to that treatment. Thus, continuing that treatment becomes unlawful,^30^ as there is no basis on which the court can rest its consent. It should be noted here that the language used bears out the reality of the position of the English courts; that they are declaring the doctors’ proposed course of action to be lawful, rather than imposing their own decision as to treatment.

Charlie’s parents, however, appealed^31^ on the grounds that the judge had failed to appropriately balance the benefits and burdens, and also that, where parents proposed alternative viable treatment, the court could only overrule if it would be likely to cause significant harm to the child, and that the usual best interests test did not apply in this context. However, not all cases involve factors which can be so balanced;^32^ since there was no longer any treatment which could offer any benefit, there was simply nothing which required to be balanced against the burdens of continued treatment.

The case of Alfie Evans and his parents’ determined attempts to secure continued treatment has a lengthy judicial history. The case came to court following irreconcilable differences between the hospital (proposing withdrawal of futile treatment and provision of palliative care only) and his parents (proposing relocation to another facility and continued treatment). Hayden J, in the High Court,^33^ relied on a legal framework for decision making which he described as both easy to state and hard to apply: in making these decisions, the best interests of the child are ‘the lode star which guides the Court’s approach’.^34^ He concluded that ‘the continued provision of ventilation, in circumstances which I am persuaded is futile, now compromises Alfie’s future dignity and fails to respect his autonomy. I am satisfied that continued ventilatory support is no longer in Alfie’s best interests.’^35^ Since it was no longer lawful for it to be continued, it would be both lawful and in his best interests for him to be extubated and given palliative care. Subsequent hearings drew no more favourable conclusions in the Court of Appeal, the Supreme Court or the European Court of Human Rights.^36^ The case returned to the High Court, but only to determine an appropriate end of life plan, which Hayden J endorsed. At the same time, he also dismissed a *habeus corpus* application, and a subsequent further appeal to the Supreme Court and application to the European Court of Human Rights were rejected, as were subsequent separate applications for leave to appeal by both parents.^37^

### B. ‘Significant Harm’ and the Relevance of Parental Autonomy

An argument based on significant harm was raised *de novo* on appeal in *Gard*.^38^ It relied on the concept of parental autonomy and the right of parents to make treatment decisions for their child. It was argued that if parents proposed a viable alternative treatment, then it must be preferred unless it was likely to cause significant harm to the child. This significant harm test comes from *In Re King*,^39^ where the parents put forward a different type of radiotherapy treatment from that proposed by the hospital. They removed their child from the hospital and headed to Spain, where they were arrested and the child was returned to the UK and made a ward of court. Baker J held that parents have responsibility for deciding on treatment for their children without interference, unless the child is suffering or is likely to suffer as a result of receiving care which no reasonable parent would give their child. This test is in line with the Children Act 1989, section 31, which applies ‘significant harm’ as the threshold for placing a child in local authority care.

In rejecting this argument in Charlie’s case, it was held that ‘the sole principle is that the best interests of the child must prevail and that must apply even to cases where parents, for the best of motives, hold on to some alternative view’.^40^ The court made it clear that *In Re King* was a very different case, where the parents were proposing other treatment which was on a roughly equal footing with that proposed by the doctors. In such cases, the court could properly allow parental autonomy to take precedence. However, Charlie’s parents were not proposing a course of treatment which could amount to a viable alternative, and thus there was no basis for invoking *King*, or for supplanting the best interests test.^41^ The decision that nucleoside therapy was unlawful ‘result[ed] from a 100%, child focused, court-led evaluation where the one issue was whether or not the therapy was in the child’s best interests’.^42^

Alfie’s parents, like Charlie’s, also attempted to raise an argument based on the significant harm test. Their position was that the ‘best interests’ of Alfie was not the determining test but that the proper determination was whether their plans to continue treatment elsewhere would be likely to cause him significant harm. This issue had not been raised before Hayden J and relied on an analogy between Alfie’s situation and section 31 of the Children Act 1989, which applies a significant harm test in care proceedings as a barrier to over-hasty removal of a child from its parents. Significant harm operates as a threshold to justify state interference in what is properly the family domain, but this only applies in care and removal cases, which are of a quite different species. In proceedings related to the withdrawal of treatment from a child, it was found that the ‘best interests test’ (described as the ‘gold-standard test’)—must be applied.^43^

The argument for using significant harm as the appropriate threshold here is put forward at least in part because it is felt that it takes due account of the realm of privacy which exists within the family context, and the extent to which parents are the best arbiters of the wider aspects of decision making for children. This encompasses more than the instant decision; it also includes a breadth of other value decisions which reflect that particular family and its beliefs and values.^44^ This is further reinforced for proponents of the significant harm test by the extent to which allowing this greater degree of parental autonomy operates to encourage and entrench the pluralism which exists within our society, and gives effect to the liberal values of freedom of expression, religion and the right to privacy itself. These freedoms and rights are best upheld, it is argued in this context, by leaving decision making within the sphere of the family, and in the hands of those with parental authority over the child.^45^ Auckland and Goold cite Goldstein^46^ here, asserting that, in situations where reasonable individuals can and do disagree, the existence of that reasonable disagreement between them means there is no objectively right or wrong approach. The assertion is, again, that parents are the best decision makers in these situations, unless their decision would raise a serious risk of significant harm.

Auckland and Goold apply this to *Gard*, which they classify as a case about balancing the potential harm of undergoing the parents’ desired experimental treatment, against the possible benefits Charlie might gain, and that since decisions about what is best for the child often revolve around values, parents (who are said to be best placed to decide which values are of most significance to them as a family unit) are the appropriate decision makers. In adopting this position, the authors also assert that such an approach affords us the means to both recognise and protect differences in views and values, which should be tolerated.^47^ However, the countervailing view, and the view expressed by the court in *Gard* in adhering to the best interests test, is that Charlie’s situation is precisely not one in which harm and benefit can be balanced.

In rejecting this ‘balancing’ approach, the court made it clear that they could not balance the potential benefits of the experimental treatment against the potential burdens, because the experimental treatment was incapable of achieving any improvement in Charlie’s condition, and therefore all that remained was the burden imposed on him by continued life support. This conclusion then justified the court in taking decisional authority away from the parents and using the best interests test to set out what the court determined to be the appropriate course of action. Auckland and Goold also raise cases of factual disputes between parents and doctors as a situation where the significant harm test should be employed in place of the best interests test, unless and until the parents’ decisions risk significant harm to the child.^48^ They argue that parents routinely make errors of fact in respect of decisions they take for their children, but that in most cases these are not sufficiently serious to justify overriding the privacy otherwise afforded to the family.

While this argument may hold for many situations, and may also hold for situations involving disputes as to which of two broadly equal treatments should be undertaken, it cannot hold for cases such as *Gard*, where the errors of fact disputed before the High Court related to the parents’ belief in the potential efficacy of an experimental treatment which was unsupported by the scientific evidence. Their error here would not be something which would have minimal impact on their child’s life, as continuing life support in order to move Charlie so he could begin the experimental treatment would have harmed him as it would have amounted to futile treatment, with unknown risks and side effects, which had no prospect of offering any benefit.

Gollop and Pope put forward some contrary views, to counter suggestions that the significant harm test would provide a better outcome in these cases.^49^ Referencing *R (A Child)*,^50^ which involved the local authority using care proceedings and the significant harm test as it appears in section 31 of the Children Act 1989, they highlight the potential ‘chilling effect’ of the significant harm test. In *R*, the child’s mother had ultimately refused consent for antibiotics to be administered, which precipitated the care proceedings. In setting out their case under section 31, the local authority had pulled together every instance of disagreement between her and the doctors, and every refusal she had made up to that point, to strengthen their case. Gollop and Pope contend that this poses real risks for parents, were the significant harm test to be adopted into medical decision-making cases, as it might well leave parents feeling they cannot stand up for what they believe to be in the best interests of their child, for fear that each time they do so, it would be reported to the local authority under the guise of safeguarding the child, and that parents would therefore effectively build a case against themselves as having caused significant harm.

Within the relevant English and Welsh legislation, conceptions of parenthood are founded on the parents’ normal rights (and duties) to care for and decide for their children, to protect their welfare and interests, and involve a blend of rights over, balanced by responsibilities for, the child.^51^ In the face of any challenge to this parental authority, whether that comes from doctors holding contrary views as to treatment (and, by implication, what treatment is or is not harmful^52^) or the court stepping in to determine the child’s best interests, it is understandable that this affront to parental control could become a battleground, but it is important here to remember that none of the arguments put forward in favour of the best interests approach above, and in both *Gard* and *Evans*, involve the complete elision of parental views. Those views are important for many reasons, not least some of those highlighted by Auckland and Goold in favour of the significant harm test (the wider familial context, beliefs and values), but the ‘gold standard’ of the best interests test demands a child-centric approach, which has to mean that parental views become part of the overall picture, rather than determinative of the decision to be made. Recognition of the fact that parents will not find this an easy position to accept is implicit in Francis J’s judgment in *Gard*, where he concludes with a plea for some form of mediation to attempt to avoid adversarial proceedings.^53^

The debate concerning ‘significant harm’, in this context, affords little guidance to Scots lawyers. The direction in which English law has developed is not mirrored in Scotland, despite the view that medical law permeates the jurisdictional border intact. The legislative basis for the ‘significant harm’ test, section 31 of the Children Act 1989, does not apply in Scotland and it cannot be presumed that, in the absence of comparable legislation, the Scottish courts would develop Scots law in lockstep with England.^54^ Nevertheless, the concept of ‘best interests’ does indeed operate in Scots law in like manner to English, as indicated by the case of *Finlayson, Applicant*.^55^ In this case, the parents of a nine-year-old haemophiliac child had refused consent to the standard treatment for that disease. The court overruled the decision of the parents on the grounds that this refusal of treatment was not in the ‘best interests’ of the child; such a refusal of treatment was deemed likely to cause unnecessary suffering and/or seriously impair the child’s health or development.

The context of *Findlayson* is quite different to that of *Gard* and *Evans*, however. Primarily, this case was concerned with aspects of social work and care proceedings under a Children’s Hearing—the adjectival law governing which is quite distinct from the laws of procedure that would govern any case concerning the withdrawal of medical treatment from a patient in Scotland.^56^ Although the case invoked the ‘best interests’ test, ultimately the judgment contains no further discussion of parental autonomy or the right of the court to intervene. The importance of the concept of ‘parental autonomy’ in cases involving children cannot be overstated; as discussed below, in most Western jurisdictions,^57^ the law takes the principle of ‘patient autonomy’ as a principal starting point in medico-legal matters and, where the patient themself lacks capacity to make decisions, the law must make provision to determine who (or which body) ought to be empowered to make decisions on behalf of the *incapax*. As will be seen in what follows, the decision of the court under *parens patriae* in Scotland would involve taking a significantly different approach to the relative standing of clinicians and parents.

### C. Decision Making: The Principle of Autonomy v Best Interests

Cases such as *Gard* and *Evans* show how theoretical niceties can become infinitely more difficult in practice, particularly in cases involving heightened emotions. However, those theoretical niceties require some discussion in the context of understanding the court’s role and approach to decision making. Decision making falls into two broad camps: one based on the exercise of autonomy by a competent adult and one based on the application of the best interests test for those who are, or have always been, incompetent. Both have generated significant case law and resulted in the spillage of a good deal of academic ink. And yet, human experience, and the frontiers of medical innovation, continue to generate novel issues to trouble courts and commentators. Determining an appropriate course of action in the case of a terminally ill infant, whose parents and doctors fundamentally disagree, will present such an issue for the Scottish courts when it (inevitably) arises this side of the border.

A fundamental aspect of medical law revolves around the definitions of both the process of decision making and the identity of the decision maker. Recognising the value of the concept of ‘autonomy’, in common law and civilian jurisdictions alike, competent adults are recognised as holding the right to be the sole arbiter of treatment decisions.^58^ ‘Autonomy’ finds its first expression (as *autos nomos*, or self-rule) as a political concept applied to the independent city states within Ancient Greece,^59^ and has a significant role in, among others, Kantian conceptions of free will and moral agency,^60^ but this lies outside our present scope. In bioethics, autonomy has often been viewed as sitting at the apex of four principles in Beauchamp and Childress’s concept of principlism.^61^ While it is often viewed as *primus inter pares*,^62^ the authors were clear that no single principle overrides the others.^63^ That caveat aside, it is clear that autonomy has developed a strong position in modern healthcare and ethics, and has driven a discourse with the individual/patient as decision maker. Autonomy is therefore often defined as the ability to exercise free choice over the course of one’s decisions and one’s body. This requires that the individual has the capacity to make meaningful choices, and is free from external influences which impact that choice. To be autonomous, actions must be voluntary. This involves a level of freedom from influence which withstands the pressures that others might bring to bear on the individual. Beauchamp and Childress offer a three-condition approach to autonomy which requires that the individual is capable of an intentional act, has a substantial degree of understanding (because requiring *perfect* understanding would rob most of us of autonomy most of the time), and a lack of external influences or internal mental conditions which would otherwise limit that voluntary choice.^64^ Thus, the ability to be self-determining feeds directly into legal conceptions of capacity.

In England and Wales, the Mental Capacity Act 2005 defines incapacity as the inability to make a decision because of mental impairment or dysfunction, and, further, as a state in which the individual cannot understand, retain, use or weigh information in order to come to a decision, or as an inability to communicate that decision.^65^ The Adults with Incapacity (Scotland) Act 2000 defines incapacity as an inability to act, or make, communicate, understand or retain a memory of a decision.^66^ The principle of autonomy in its guise as a right to self-determination also infiltrates established human rights, and statements are made within Strasbourg jurisprudence in the context of the interpretation of the right to respect for one’s private life under Article 8 of the European Convention on Human Rights, to the effect that

Though no previous case has established as such any right to self-determination as being contained in Article 8 of the Convention, the Court considers that the notion of personal autonomy is an important principle underlying the interpretation of its guarantees.^67^

Within common law jurisprudence, there are numerous examples wherein the courts have upheld the decision of the individual on those very grounds of autonomy and self-governance. Such examples are not limited by jurisdiction.^68^ Thus, in America, Cardozo J states that ‘Every human being of adult years and sound mind has a right to determine what shall be done with his own body’,^69^ and in Canada, Cory J stated the following:

It should not be forgotten that every patient has a right to bodily integrity. This encompasses the right to determine what medical procedures will be accepted and the extent to which they will be accepted. Everyone has the right to decide what is to be done to one’s own body. This includes the right to be free from medical treatment to which the individual does not consent.^70^

In England, the importance of capacity finds expression in *Re T (adult: refusal of medical treatment)*,^71^ where the hospital was granted an order to give a blood transfusion to a patient who had refused such treatment in writing, prior to deteriorating to the point where emergency intervention was necessary. The court found that she had refused treatment under the influence of her mother. It was therefore able to find that her refusal of treatment was not a genuine exercise of her autonomy, and that her medical condition rendered her incapable of making a valid refusal of life-saving treatment. Notwithstanding the decision in this case, the judgment makes the *competent* adult’s right of refusal abundantly clear. So we find Lord Donaldson asserting that ‘If the patient had the requisite capacity, [doctors] are bound by his decision. If not, they are free to treat him in what they believe to be his best interests’,^72^ and that ‘the patient’s right of choice exists whether the reasons for making that choice are rational, irrational, unknown or even non-existent’.^73^ He went on to state that ‘*Prima facie* every adult has the right and capacity to decide whether or not he will accept medical treatment, even if a refusal may risk permanent injury to his health or even lead to premature death’.^74^

This reasoning was echoed in *Ms B v An NHS Hospital Trust*,^75^ which involved a request from a patient, who had the requisite ‘capacity’, to withdraw ventilation, even though such would almost certainly lead to her death. Lady Butler-Sloss affirmed that ‘Unless the gravity of the illness has affected the patient’s capacity, a seriously disabled patient has the same rights as the fit person to respect for personal autonomy’.^76^ Lord Goff stated, in relation to a competent adult’s refusal of treatment, that the doctors must follow the individual’s wishes, no matter how unreasonable the refusal.

To this extent, the principle of the sanctity of human life must yield to the principle of self-determination and, for present purposes perhaps more important, the doctor’s duty to act in the best interests of his patient must likewise be qualified.^77^

Thus, medical practitioners are obliged to respect any decision to refuse treatment made by competent patients, even where that decision seems unusual, if not downright bizarre, to others,^78^ and even if it will precipitate their death.

While the principle of autonomy may justify the legal position as expressed in *Ms B*, it provides little guidance in cases concerning decision making for individuals who lack capacity. Infant children clearly lack decision-making capacity^79^ and so, in such cases, the assumption is that the parents (biological or legal) will share a decision-making role with the child’s clinical team and jointly make decisions with that team on the basis of the ‘best interests’ of the child.^80^ This forms a necessary exception to the modern pendulum swing towards individual patient autonomy and leaves decision making for children and others who lack capacity firmly within traditional paternalism. The basis for subsequent court intervention in such cases arises solely where clinicians and parents cannot agree on a course of action. Given the nature of these cases and the reasonably urgent time frame involved, there is generally a pressing need for ‘a’ decision to be made.

Lord Donaldson’s decisions resonate through the English case law of the 1990s. In *Re C (a minor) (withdrawal: medical treatment)*,^81^ the court, concurring with his judgment, allowed the hospital to withdraw treatment, based explicitly on considerations of that child’s welfare, well-being and best interests. Little over a year later, in *Re J (A Minor) (Wardship: Medical Treatment)*,^82^ Lord Donaldson noted that ‘there will be cases in which … it is not in the best interests of the child to subject it to treatment which will cause it increased suffering and produce no commensurable benefit’.^83^ His Lordship went on to set out several key points for consideration in cases of this kind: that there was a presumption in favour of life, but that it must be considered from the assumed position of the patient; that decision making is a cooperative effort between doctors and parents (or courts, in cases of wardship), where all decisions must be made in the ‘best interests’ of the child; and that no decision taken on the basis of ‘best interests’ should be considered as one *designed* to bring about death, other than as a side effect.

The English courts have deliberately left the definition of ‘best interests’ unclear.^84^ Although it has been emphasised that no attempt should be made to lay out specific criteria for the determination of ‘best interests’, it has been recognised that the determination of ‘best interests’ must be judged from the assumed perspective of that child (or other *incapax* person), with a strong but rebuttable presumption in favour of life. In making any decision to withdraw or continue treatment of children, the courts have emphasised that the welfare of the child is key. The pattern developed by the courts in cases of this kind is brought out in *An NHS Trust v MB.*^85^ There is no role in such cases for either substituted judgment or assessments of what the reasonable doctor or parent would do.^86^ All cases stand on their own facts. The views of doctors and parents must be carefully considered, but are not relevant to the test, centred as it is on the patient’s ‘best interests’.

What constitutes the child’s ‘best interests’ is determined by the court, using its own objective judgment, with ‘best interests’ being understood in its widest sense, including medical, emotional, sensory and instinctive^87^ considerations. The court in *MB* recognised that taking a ‘balance sheet approach’ can help; this involves looking explicitly at the relative benefits and burdens of the treatment, while simultaneously acknowledging that there can be no computational basis for assessing their relative weights.^88^

The importance of finding the balance between benefits and burdens is well illustrated by *An NHS Trust v A and B and another*,^89^ involving a dispute between doctors and parents over the continued treatment of a severely brain-damaged infant, for whom there was no possible treatment and for whom death was inevitable. The Trust had made an application to the court for a declaration that it would be lawful and in his best interests for him not to be ventilated and resuscitated, and for his treatment to be limited to palliative care. His parents wanted further trials of drugs to combat his seizures, along with continued ventilation and CPR. The medical evidence was clear that there was no possible benefit from any further drug treatment, and that re-ventilating and resuscitating him caused pain and distress. Relying on *Re T (Wardship: Medical Treatment)*,^90^ Russell J determined that it was within the power of the English courts to overrule the parents in accordance with the guidelines set out in *Portsmouth NHS Trust v Wyatt*,^91^ which focus on an objective assessment of best interests, balancing welfare in the widest sense with the presumption in favour of life, looking from the assumed position of the patient. In balancing the burdens of his untreatable seizures, and the pain and distress involved in his ultimately futile current treatment, against the minimal level of comfort he appeared to derive from contact with his parents, Russell J concluded that it was in the child’s best interests, and lawful, for the treatment to be withdrawn, and for his life to come to as comfortable an end as possible.

It should be noted that there is an evident reluctance by the courts to rule against the decision of a doctor, if to do so would require them to treat their patient contrary to their clinical judgment, as can be seen from *Re J (a minor) (child in care: medical treatment).*^92^ The net effect of this judgment must be that, while the explicit position is that the court decides, there are few instances where the doctor’s decisions are not, in reality, determinative. A reluctance to decide a case so as to force a doctor to treat against his clinical judgment must surely mean that medical considerations carry greater weight. If, for example, medical considerations determined that treatment was not in the child’s best interests, but non-medical factors on the balance sheet indicated that continued treatment would be in the child’s best interests, the authority of *Re J* would demand that the balance be reversed in favour of the doctor’s assessment, because to do otherwise would require the courts to force the doctor to treat the child against his clinical judgment. In Scotland, however, these matters are dealt with by parental fiat. The doctor’s role is to persuade the parents of the efficacy of his proposed course of action. The ‘parents’ may be the biological or legal parents, failing whom the court, acting as *parens patriae*.

Due to these differences in procedural law, the hands of the Scottish courts are more constrained than those of the English courts. In Scotland, it might not be possible for the Court of Session to give effect to the will of the doctors because the court must do more than simply declare their proposed course of conduct lawful. As is elucidated below, since the court must act as though it were the patient or the parent of the patient, the Scottish courts are obliged to give due consideration to the subjective will of the patient (if such can be determined): thus, it might be concluded that Scots law gives more weight in these circumstances to parental autonomy than would be the case south of the border.

## 3. The Scottish Legal Position

### A Looming Issue: Whither Scots Law?

A.

The adjectival law which would regulate a case such as *Gard* or *Evans*, should similar facts confront the Court of Session, differs materially from the procedural law that governs the English High Court.^93^ Indeed, the substantive law of Scotland, and the rationale underpinning it, also differs from the positions expressed by Francis J and Hayden J in *Gard* and *Evans*. It is not competent for a physician to seek a declarator from the Outer House of the Court of Session that their proposed course of conduct is lawful. Rather, the physician must directly petition the Inner House of the Court of Session to use its inherent *parens patriae* jurisdiction and consent on behalf of the patient, as if the court itself were the parent.^94^ This *parens patriae* jurisdiction might apply in cases concerning incompetent adults or infants^95^ and has a long history, but has not received much consideration in Scottish legal literature.^96^

Historically, the Kings of both Scotland and England held *parens patriae* jurisdiction over their subjects.^97^ The emergence of the *parens patriae* jurisdiction in Scots law has not been studied in any great detail,^98^ but it is clear that by the 16th century the Scottish monarch was expressly given the care of the helpless ‘when agnates [ie male lineal blood relatives] fail’.^99^ This exercise of *parens patriae* practically occurred in the appointment of tutors dative, on the authority of the King, through the King’s Court of Exchequer.^100^ Though the Court of Session could also, by the 18th century, concurrently appoint tutors by exercising its *nobile officium*^101^ (that is, the ‘extraordinary equitable jurisdiction of that court’^102^), as a strict matter of law,^103^ appointments of tutors dative were still said to be passed ‘by the King alone, as *pater patriae*’.^104^

By the mid-19th century, ‘the whole power, authority, and jurisdiction’ of the Court of Exchequer in Scotland was transferred to the Court of Session.^105^ Since it was already possible for the Court of Session to appoint tutors dative by exercise of the *nobile officium* even prior to this, it has subsequently been posited that the *parens patriae* power of this court is now little more than an applied exercise of the *nobile officium*.^106^ In his monograph on the *nobile officium*, however, Thomson notes that, although this position generally suffices in matters of practice,^107^ it remains conceptually clear that *parens patriae* is of a far narrower remit than the more general equitable jurisdiction of the Court of Session.^108^ The conflation of *parens patriae* and *nobile officium* is, however, understandable, due to the judgment in *Stuart v Moore*.^109^ In this case, the Lord Chancellor suggested that the Court of Session’s *nobile officium* jurisdiction was conferred upon it by the Sovereign, who stood as *parens patriae*,^110^ and this jurisdiction gave the court a ‘duty to take care of all infants who require their protection, whether domiciled in Scotland or not’, emphasising that ‘the benefit of the infant is the foundation of the jurisdiction, and the test of its proper exercise’.^111^

Such does not appear to accurately reflect the nature of the *nobile officium*,^112^ which, as argued before the Inner House the previous year, was invoked in *Stuart* in a peculiar sense, as a ‘jurisdiction that might be exercised to aid in giving effect to the orders of a foreign Court’,^113^ being that the Court of Chancery was and is foreign to Scotland.^114^ Indeed, it appears (as indicated in the English, but not the Scottish, case report) that the Lord Chancellor’s judgment was not properly concerned with the nature of the *parens patriae* jurisdiction of the Court of Session at all, but rather with the more general point that both the Supreme Courts of Scotland and the English Court of Chancery, ‘representing the Sovereign as the *parens patriae*, were bound to assist each other in doing what was necessary to ensure the benefit of the infant [in *Stuart*]’.^115^ The Lord Chancellor appears to have been expressing two distinct points: that the authority of the court descends ultimately from the Sovereign, and a more specific statement about the application of the *nobile officium* to the case at hand.

In the same case, Lord Cranworth more clearly explains the nature of the *parens patriae* jurisdiction within Scotland.^116^ Though he noted that this jurisdiction might sometimes correlate with the *nobile officium*, as it did in *Stuart*,^117^ it was said that ‘the Crown [is] *parens patriae*, and protector, therefore, of infants who have had the misfortune to lose their parents’.^118^ Since the Court of Session was recognised as possessing a *nobile officium* (ie equitable) jurisdiction which ‘corresponds very much to that which exists in the Lord Chancellor in this country [ie in England]’,^119^ it was possible for the Scottish court to uphold the judgment of the English court by utilising its *nobile officium* and—in the circumstances of the case, given that the benefit of the infant was paramount^120^ and that the House of Lords had ruled that it would be in the interests of the child for the English judgment to be executed^121^—the Supreme Courts of Scotland were obliged to make use of this mechanism.^122^ Thus, *Stuart* recognised that the *nobile officium* was merely, as an equitable mechanism, one possible means of fulfilling the court’s duty to exercise *parens patriae* jurisdiction; *parens patriae* and *nobile officium* cannot be said to be, therefore, one and the same.

Accordingly, it appears clear that the Court of Session presently has *parens patriae* jurisdiction not as a part of its *nobile officium*, but rather due to its inheritance of the ‘whole power, authority and jurisdiction’*—*including *parens patriae—*of the Court of Exchequer in Scotland.^123^ Thus, *nobile officium* follows from the court’s authority to enact the decisions of the *parens patriae*, not vice versa. Though the power of *parens patriae* over adults was withdrawn in England and Wales by the Mental Health Act 1959, the relevant provisions of this Act did not extend to Scotland.^124^ The Mental Health (Scotland) Act 1960 fulfilled much the same role in Scots law, ‘sweeping away’, as stated in *Re: F*,^125^ all previous ‘lunacy’ legislation. The Scottish Act, however, did not alter the inherent powers of the Court of Session.^126^ Thus, the Scottish courts’ exercise of *parens patriae* jurisdiction was not affected by the 1960 Act and so *parens patriae* remains of relevance to Scots lawyers today in cases of adult persons of unsound mind, as well as in cases of tutory.^127^

The continuing relevance of *parens patriae* was affirmed in 1996, in *Law Hospital NHS Trust v Lord Advocate*,^128^ where the Court of Session ruled that the jurisdiction extended to incapable adults, as well as those patients who are in a persistently vegetative state (PVS).^129^ This decision, in line with the English decision of *Airdale NHS Trust v Bland*,^130^ held that the withdrawal of artificial hydration, nutrition or non-palliative treatment may be in the ‘best interests’ of a PVS patient.^131^ In recognition of this, the court utilised its *parens patriae* jurisdiction to authorise the withdrawal of treatment and, in doing so, noted that, functionally, ‘authorisation in the exercise of [*parens patriae*] jurisdiction [has] the same effect in law as if consent had been given by the patient’.^132^

The court in *Law Hospital* noted that they ‘were not referred to any Scottish case in which the *parens patriae* jurisdiction [had] been exercised in this way [ie as a means of authorising the withdrawal of medical treatment]’.^133^ Similarly, there was ‘almost no guidance in the Scottish authorities, such as they are, relating to the exercise of *parens patriae* jurisdiction with regard to the test to be applied in deciding whether or not a course of conduct should be authorised’.^134^ Nevertheless, the Inner House determined that the *parens patriae* jurisdiction could be utilised as requested by the pursuers^135^ and that the test to be applied was, as elsewhere,^136^ that of ‘best interests’.^137^

The language of ‘best interests’ echoes the opinion in *Stuart v Moore* that the *parens patriae* jurisdiction of the Court of Session behoves judges to do what is ‘necessary to ensure the benefit of the infant’, that being ‘the foundation of the jurisdiction’. The idea of the ‘benefit of the [*incapax*]’ thus correlates with the concept of ‘best interests’ as it operates within Common law jurisdictions; indeed, the *corpus* of authority concerning such was drawn upon by the Inner House in *Law Hospital*. Accordingly, the decision of the court in *Law Hospital* merits deeper consideration.

### B. Law Hospital NHS Trust v Lord Advocate

The facts of *Law Hospital*, at first sight, do not concern withdrawing treatment from a terminally ill infant, although the case nevertheless represents the last word, in Scotland, on the subject of withdrawal of medical treatment from incapable patients. In *Law Hospital*, it was held that a declarator that the conduct of the physicians would be deemed lawful was thought to be appropriate in the case;^138^ however, the court stressed that subsequent complex cases should proceed by petition to the *parens patriae* jurisdiction of the Inner House, and that declarators in such cases would be inappropriate.^139^ There have been no such reported petitions to this court since *Law Hospital* in 1996, however.

At the time that the *Law Hospital* case went to court, the patient had been in a persistent vegetative state for four years.^140^ All her surviving relatives agreed that treatment should be discontinued.^141^ The Lord Advocate did not oppose this;^142^ he appeared only as ‘defender in the public interest’.^143^ The dispute was not between the family members and the clinicians; rather, the central issue was said to be one of procedural law tinted with ethical concerns.^144^ The Inner House recognised that ‘court procedures differ in many respects from those in England, and materially so in the present context’.^145^ These differences did not result in a different practical outcome from the comparable English case of *Bland*,^146^ but the reasoning of the court was necessarily distinct.^147^ Noting that the ‘wardship jurisdiction is, in modern form, the exercise of the *parens patriae* jurisdiction formally vested in the sovereign’,^148^ the Court of Session referred to the English case of *Re: B*,^149^ in which the ‘primary and paramount consideration’ was held to be the welfare and best interests of the ward.^150^ Recognising that ‘the same test is [also] being adopted where the *parens patriae* jurisdiction is not now available’,^151^ (then) Lord President Hope held that the law of Scotland ‘should approve of the application of that [the ‘best interests’] test in such cases where the issue is whether a tutor-dative should be authorised to consent to medical treatment [or indeed withdrawal of treatment] of the ward’,^152^ on the basis that

it would be unsatisfactory if the court were to be required, in the exercise of [*parens patriae*] jurisdiction, to apply different tests according to the circumstances of each case … The better course is to recognise that all these cases require to be decided by reference to the same fundamental principle.^153^

Accordingly, although *Law Hospital* was concerned with an incapable adult, since the decision was predicated on the exercise of *parens patriae* jurisdiction, the principles are also relevant in determining the law on the withdrawal of treatment from terminally ill infants. Referring to an unnamed Irish Supreme Court case,^154^ the Court of Session determined that there was no principled reason why it should not ‘decide what is for the benefit of persons [who are of nonage, or mentally incapable] and thus incapable of taking decisions for themselves’.^155^ As Lord Clyde indicated,^156^ the court felt that proceeding on the basis of providing authorisation on behalf of the *incapax*, rather than on permitting the withdrawal of treatment on the basis of declarator, had distinct advantages.^157^ Procedurally, this was not least because a declarator from the civil court could not lawfully bind either the Lord Advocate or the High Court of Justiciary^158^ and, ultimately, because ‘the consequences of permitting the civil court to determine directly matters properly falling within the separate jurisdiction of the criminal court could lead to some considerable confusion’.^159^

In respect of the criminal dimension of *Law Hospital*, the Lord Advocate had undertaken to ‘make a statement on his policy as to whether or not to prosecute’,^160^ and this was promptly issued,^161^ indicating that, although there was no impetus in the civil law to petition for authorisation in every case, the Lord Advocate would only guarantee that no prosecution would follow where the Court of Session had provided such authorisation.^162^ In a paper delivered to the Royal College of Physicians and Surgeons of Glasgow (in an ‘unofficial ambience’)^163^ soon after the policy statement, the Lord Advocate stated that ‘it is for doctors and relatives involved in such tragic situations to decide which course of action they should adopt’.^164^ Thus, as Laurie, Harmon and Porter observe:

both the Lord Advocate and the Lord President of the time agreed that decisions could be made on medical grounds or independently of the courts—but neither gave any guidance as to when it would be either necessary or unnecessary to seek judicial approval.^165^

Although prosecutorial policy, if not the letter of the civil or criminal law,^166^ became unclear after *Law Hospital*, no Scottish court to date has prosecuted a physician for withdrawing treatment. This is so despite the fact that, similarly, there has been no reported case concerning a petition to the Court of Session’s *parens patriae* jurisdiction,^167^ yet ‘if a person does something which he knows will cause the death of another person, he will be guilty of homicide if his act is the immediate and direct cause of the person’s death’.^168^ Though the newly reconstituted Scottish Parliament passed the Adults with Incapacity (Scotland) Act in 2000, this did not change the position of the criminal or civil common law on the withdrawal of treatment.^169^ Indeed, the (then) Scottish Executive stressed that withdrawal and withholding decisions were outwith the purview of the legislation.^170^ The Scottish medical and legal professions have, therefore, operated—in cases concerning both adults and infants—on the basis of a generous interpretation of *Law Hospital*. As a matter of practice, ‘the decision as to whether an application is necessary [rests] in each case with those who will be responsible for carrying that intention into effect’^171^ (which in cases concerning PVS patients and terminally ill infants will invariably be the treating clinicians). Thus, treating clinicians—if unopposed by the patient’s relatives^172^—in fact have a free hand (on the basis of ‘indefensibly vague’ limitations)^173^ to determine the outcome of such cases.

In *Law Hospital*, Lord Milligan explicitly called for legislation on this issue, recognising that ‘the need for legislation concerning the substance of authorisation is the same in Scotland as in England’.^174^ None has been forthcoming from either jurisdiction, despite the re-establishment of the Scottish Parliament just a few years after this case. In the absence of legislation, and any further Scottish case law concerning withdrawal of treatment, the common law as expressed in *Law Hospital* continues to apply in Scotland. As such, as a matter of public policy rather than of law, it appears that the Scottish medical profession has been granted an exceptional privilege to determine what is in the ‘best interests’ of those under its care, and to act accordingly, without much legal oversight or interference.

This is problematic, not least because the Inner House in *Law Hospital* expressly refused to rule on the question of criminal culpability. Though it might be hoped that ‘good common sense would prevail’ in even difficult cases,^175^ it seems naive to do nothing more than trust in the wisdom of those who hold high office. The possibility of the Crown Office initiating a prosecution of any physician who has acted without approval from the Court of Session remains open, even if it might be unlikely. Likewise, though private prosecution in Scotland is extremely rare,^176^ it cannot be presumed that parents, faced with the prospect of losing a child,^177^ might not push for a private prosecution, or (perhaps more likely) for civil redress.

### C. Gard, Evans *and the Court of Session*

But for one complication,^178^ at common law, there would have been no justiciable conflict between the physicians and parents in any Scottish analogue to *Gard* or *Evans*, since *patria potestas* was vested in the father in respect of his legitimate children.^179^  *Patria potestas* historically carried with it ‘powers of custody, residence, education, general upbringing, religious training, legal representation and medical treatment’.^180^ Accordingly, fathers made the ultimate decision as to all medical treatment (and, by inference, withdrawal of treatment).^181^ The Scottish courts, then, would have been forced to yield to the expressed wishes of the father;^182^ there would have been no scope for the *parens patriae* jurisdiction to be invoked.^183^ In the words of Wilkinson and Norrie, the authors of the leading Scottish text on the law of parent and child:

The father, it was held, was the best judge of what was in the interests of the child. The court interfered where the child’s welfare was seriously endangered by the father’s conduct, not because the law was otherwise neglectful of the interests of the child but because, unless there was clear evidence that the father was abusing his position, interference by the court would be to substitute an inferior for a superior view of where those interests lay.^184^

Absent statutory enactment, it is clear that *parens patriae* jurisdiction could be invoked only ‘where agnates fail’.^185^ With the introduction of the Guardianship Act in 1973, mothers also acquired *patria potestas*,^186^ but by then the power had been significantly diluted.^187^ The power conferred on mothers is nevertheless such that they too would enjoy the normal powers of parenthood, meaning that the *parens patriae* jurisdiction can only be exercised not simply where agnates fail, but, indeed, where all cognates fail.

By the 1990s, the control granted by *patria potestas* had entirely ceased to be judicially recognised and the common law had been largely replaced by statute.^188^ At present, since ‘it would be open to the Court of Session in exercise of its *parens patriae* jurisdiction to make orders authorising or prohibiting medical treatment’,^189^  *parens patriae* now holds supremacy over any residual *patria potestas*. In a complete reversal of the common law position expressed by Wilkinson and Norrie,^190^ in the ‘rare and exceptional’^191^ cases in which parents disagree with physicians as to the question of withdrawing treatment, the view of the father (and mother) may now be expressly deemed to be inferior to that of the court. Thus, were a case akin to *Gard* or *Evans* to arise in Scotland, it would be competent for the physicians to petition the Court of Session to exercise its *parens patriae* jurisdiction and substitute its own decision—which would be, in fact, the decision of the attending physicians—for that of the biological parents.

Since such a case has not yet arisen, the likelihood of the court granting such a petition cannot be definitively known. While, at one time, the Scottish courts were deferential towards decisions made by medical practitioners,^192^ which explains in large part the paucity of medico-legal cases,^193^ the attitude of judicial submission has softened even in this one-time bastion of medical paternalism.^194^ Patients are no longer seen as the passive recipients of medical care, but rather as persons—or, indeed, consumers—holding rights and exercising choice.^195^ Thus, it is open to the Scottish courts, in the 21st century, to favour the opinions of the parents of a terminally ill child, in circumstances akin to *Gard* or *Evans*, rather than to favour those of the medical professionals.

Since the Scottish courts must arbitrate between the competing views of the physicians and parents before expressly setting down its own view, as *parens patriae*, of where the ‘best interests’ of the child lie, they must have greater regard to public policy matters in the parent–child relationship. Unlike in England and Wales, it is not competent for the court to state that it is simply declaring that the physicians’ proposed conduct is lawful; the Court of Session must additionally subordinate the will of the parents to its own, should they side with medical opinion. Since ‘the contentious aspect of the case[s of *Gard* and *Evans*] is the issue surrounding parental autonomy’,^196^ this places the court in a difficult position; thus, in spite of the fact that they could now do so, the Scottish judiciary might be reluctant to utilise its *parens patriae* jurisdiction in any case in which the biological parents of the child expressed a contrary will.

## 4. Conclusion

Every lawyer is familiar with the ‘reasonable person’. It is perhaps reasonable to conclude that ‘parents, physicians and other caregivers, intimately connected through the birthing process, are the individuals best suited to make these intensely personal and wrenching decisions’ concerning the withdrawal of medical treatment from terminally ill children.^197^ The law, though, cannot presume that all parents or persons will behave reasonably under such extraordinarily circumstances. There must be judicial mechanisms in place to arbitrate between competing positions. Though the majority of cases might be practically resolved without recourse to such legal mechanisms—indeed, an additional extra-legal process of physician–parent mediation might prove useful in cases where there is a real danger of a breakdown in communication—it does not follow that there is no need to explore the letter of the law in this area. The law must be equipped to deal with the (understandably, given difficult circumstances) intractable and unreasonable person.

In Scotland, the letter of the procedural law differs materially from that in England, Wales and Northern Ireland. Rather than deciding such cases by way of declarator, the Scottish court, in *Law Hospital*, expressed that such cases should be decided by petitioning the *parens patriae* jurisdiction of the Court of Session. This jurisdiction allows the court to substitute its own decision for that of the biological parents. Similarly, though in practice physicians have enjoyed free reign to withdraw treatment if, in their opinion, it is not in the ‘best interests’ of that patient, it is likewise open to the court to refuse consent to the withdrawal of such treatment. No Scottish physician has been prosecuted for withdrawing treatment in the absence of the consent of the Court of Session. It might be inferred that this is because it is difficult, if not impossible,^198^ to prove that a physician, acting with therapeutic intent, exhibited any criminal or culpable mental state. Thus, in the absence of express determination by the courts that the withdrawal of treatment is not in the best interests of the patient, Scottish prosecutors have tended to presume therapeutic motive and abstain from prosecution. Since it is exceedingly difficult to raise a private prosecution in Scotland, it might be concluded that Scottish physicians will not be held criminally culpable for exercising their medical judgment in the absence of an express refusal of consent set out by the *parens patriae*. It is only if a physician *unsuccessfully* petitions the Court of Session, before proceeding to withdraw medical treatment regardless, that the criminal law might come into play.

The fact that the Scottish courts must exercise their *parens patriae* jurisdiction in cases concerning the withdrawal of treatment from a terminally ill infant has not proven problematic in practice, since Scotland has continued to observe its tradition of deciding medical matters privately.^199^ However, it is quite possible that the Scottish courts could, as a result of the key procedural difference discussed here, decide a case analogous to *Gard* or *Evans* differently from the English courts. This is so since the Scottish courts would be forced to expressly balance the competing interests of the parents and the physicians in deciding whether or not to exercise *parens patriae* jurisdiction. Since, historically, *parens patriae* could be utilised only if the *incapax* had no surviving (or legally recognised) parents, the Scottish courts might be reluctant to employ their *parens patriae* jurisdiction to unequivocally subordinate the views of the parents to those of the court.

With that said, since parental *potestas* has largely been replaced by a statutory regime which confers less power on parents than they enjoyed at common law, it is, of course, possible that the Scottish courts would trek a similar tack to the English courts. This does not change the fact that, though they would assess whether or not to do so with reference to the ‘best interests’ test, they would be forced to arrive at, and state, their conclusion by way of a notably distinct legal process. To speak of the topic of withdrawing treatment from terminally ill infants as a ‘UK medical law matter’ is, therefore, to err.

## Footnotes

1 *Great Ormond Street Hospital v Yates and others* [2017] EWHC 972 (Fam).

2 *Alder Hey Children’s NHS Foundation Trust Hospital v Evans and Ors* [2018] EWHC 308 (Fam).

3 Issue 44(7) of the Journal of Medical Ethics, for instance, was dedicated to the Charlie Gard case: see https://jme.bmj.com/content/44/7.

4 See Emma Cave and Emma Nottingham, ‘Who Knows Best (Interests)? The Case of Charlie Gard’ [2018] Med LR 500; Iain Brassington, ‘Alfie Evans: Please, Just Stop’ [2018] BMJ (Blog) <https://blogs.bmj.com/medical-ethics/2018/04/24/alfie-evans-please-just-stop/>.

5 For the clarity of the law, see Eliana Close, Lindy Willmott and Benjamin P White, ‘Charlie Gard: In Defence of the Law’ (2018) 44 Journal of Medical Ethics 476, 476–7.

6 For *Gard*, see George Gillett, ‘The Case of Charlie Gard Should Make Us Question Our Attitudes to Parental Autonomy’ [2017] BMJ Blog <https://blogs.bmj.com/bmj/2017/07/21/george-gillett-the-case-of-charlie-gard-should-make-us-question-our-attitudes-to-parental-autonomy/>; for *Evans*, see <https://www.bbc.co.uk/news/uk-england-merseyside-43900571>.

7 See Dominic Wilkinson and Julian Savulescu, ‘Hard Lessons: Learning from the Charlie Gard Case’ (2018) 44 Journal of Medical Ethics 438, 439.

8 This concern was expressed in academic literature by Professor Gillon: see Raanan Gillon, ‘Why Charlie Gard’s Parents Should Have Been the Decision-Makers about Their Son’s Best Interests’ (2018) 44 Journal of Medical Ethics 462.

9 See GT Laurie, SHE Harmon and G Porter, *Mason and McCall Smith’s Law and Medical Ethics* (10th edn, OUP 2016) paras 15.04–15.07; Kenneth McK Norrie, *The Law Relating to Parent & Child in Scotland* (3rd edn, W Green 2013) para 7.34.

10 Laurie, Harmon and Porter (n 9) para 15.28.

11 In the words of Laurie, ‘the importance of the best interests test as the central governing concept is common to all jurisdictions’: Graeme T Laurie, ‘Parens Patriae Jurisdiction in the Medico-Legal Context: The Vagaries of Judicial Activism’ [1999] Edinurgh Law Review 95, 101.

12 See eg Thaddeus Mason Pope, ‘Guest Editorial: Charlie Gard’s Five Months in Court: Better Dispute Resolution Mechanisms for Medical Futility Disputes’ (2018) 44 Journal of Medical Ethics 436.

13 See Craig, *Jus Feudale*, II, XX, 9; Stephen Thomson, *The Nobile Officium: The Extraordinary Equitable Jurisdiction of the Supreme Courts of Scotland* (Avizandum 2015) 118. Though itself a gender-neutral term, *parens patriae* has historically been conceptualised as the jurisdiction of the King, styled *pater patriae* (father of the nation) and, as discussed below, the jurisdiction conferred that which would ordinarily be exercised by a father (*pater*) in possession of *patria potestas* (fatherly power).

14 See *Law Hospital NHS Trust v Lord Advocate* 1996 SC 301.

15 See Eliana Close, Lindy Willmott and Benjamin P White, ‘Charlie Gard: In Defence of the Law’ (2018) 44 Journal of Medical Ethics 476, 476–7.

16 Above n 14.

17 Such parameters remain, in Scotland, ‘indefensibly vague’: see Laurie, Harmon and Porter (n 9) paras 15.121–15.127.

18 As Laurie observed in his 1999 article (n 11), while Scots law would appear to formally require the court to be called upon in each and every case to consent (or refuse) on behalf of each and every *incapax*, this does not happen in practice; in the 10th edition of *Mason and McCall Smith’s Law and Medical Ethics* (published in 2016), Laurie and his co-editors note that the consequences of *Law Hospital* were such that ‘decisions could be made on medical grounds and independently of the courts’: see Laurie, Harmon and Porter (n 9) para 15.125.

19 Indeed, it has been observed that one cannot simply ‘assume that the conception of best interests offered by the English courts is suitable in a legal system such as that of Scotland which purports to exercise the *parens patriae* jurisdiction’ (Laurie (n 11) 104), however much commonality may be assumed in practice.

20 Laurie (n 11) 101.

21 Laurie (n 11) 101.

22 In an (admittedly) English case (though the principle is, it is submitted, identical in Scotland now as it was in England then), Lord Esher MR described *parens patriae* jurisdiction as ‘a paternal jurisdiction’ which allows the court to ‘supersed[e] the natural guardianship of the parent’: see *R v Gyngall* [1893] 2 QB 232, 239.

23 Gillett (n 6); Gillet is speaking only of the case of *Gard*, but it is submitted that his statement may be read as equally applicable to Alfie Evan’s situation.

24 Infantile onset encephalomyopathic mitochondrial DNA depletion syndrome, or MDDS.

25 Above n 1.

26 *Great Ormond Street Hospital* (n 1) para 36. As is demonstrated later, overriding control is not vested in the Scottish courts in the same manner as in the English courts.

27 Including *Portsmouth NHS Trust v Wyatt* [2006] FLR 554 and *An NHS Trust v MB* [2006] 2 FLR 319. The reference to *Wyatt* in the text of Francis J’s judgment is erroneously printed as ‘[2000] 1 FLR 554’, which refers to a page midway through the report of *Re, R-B (A Patient) v Official Solicitor* [2000] 1 FLR 549.

28 For an example, see *MB* (ibid) para 60.

29 [2013] UKSC 67.

30 ibid para 22.

31 *In Re Gard (a child) (child on life support: withdrawal of treatment)* [2018] 4 WLR 5.

32 ibid 10.

33 *Alder Hey* (n 2).

34 *Alder Hey* (n 2) para 47.

35 *Alder Hey* (n 2) para 66.

36 *Alder Hey Children's NHS Foundation Trust Hospital v Evans* and Ors [2018] EWHC 818 (Fam), para 2.

37 *Evans and James v Alder Hey Children’s NHS Foundation Trust, Alfie Evans* [2018] EWCA 984 (Civ).

38 Above n 31.

39 [2014] 2 FLR 855.

40 *Gard* (n 31) 21.

41 *Gard* (n 31) 21.

42 *Gard* (n 31) 22.

43 *Evans and Ors v Alder Hey Children’s NHS Foundation Trust, Alfie Evans (by his Children’s Guardian)* UKSC 2018 WL 03440352, paras 15–16.

44 On this, see Cressida Auckland and Imogen Goold, ‘Parental Rights, Best Interests and Significant Harms: Who Should Have the Final Say over a Child’s Medical care?’ (2019) 78(2) CLJ 287, 288.

45 ibid 299–300.

46 Joseph Goldstein, ‘Medical Care for the Child at Risk: On State Supervision of Parental Autonomy’ (1977) 86 Yale LJ 645, 650; Auckland and Goold (n 44) 300.

47 Auckland and Goold (n 44) 301.

48 Auckland and Goold (n 44) 303.

49 Katie Gollop and Sarah Pope, ‘Charlie Gard, Alfie Evans and R (A Child): Why a Medical Treatment Significant Harm Test Would Hinder not Help’ <http://www.transparencyproject.org.uk/charlie-gard-alfie-evans-and-r-a-child-why-a-medical-treatment-significant-harm-test-would-hinder-not-help/>

50 [2018] EWFC 28.

51 As set out in the Children Act 1989, s 3.

52 There is a significant body of literature on this. See Douglas Diekema, ‘Parental Refusals of Medical Treatment: The Harm Principle as Threshold for State Intervention’ (2004) 25 Theoretical Medicine 243. See also Gillon (n 8) 462, where he states that ‘the law should allow parents to decide their children’s best interests unless … substantial harms or substantial injustices would result’.

53 *Great Ormond Street Hospital* (n 1) para 130.

54 See Children Act 1989, s 108 (11).

55 1989 SCLR 601.

56 For one thing, the case was decided by a sheriff (who lacks *parens patriae* jurisdiction) rather than a senator of the College of Justice sitting in the Court of Session; any case concerning the withdrawal of medical treatment would be heard by the latter, rather than the former.

57 See n 58 below.

58 The principle of autonomy can be seen to be woven through the fabric of the law in many Western jurisdictions and it has a significance far beyond that noted in the commonly cited Anglo-American cases. In Spain, for instance, the courts have recognised that a non-pecuniary (yet nevertheless compensable) harm is inflicted where a doctor proceeds without obtaining the ‘informed consent’ of their patient: see María Paz García Rubio and Belén Trigo García, ‘The Development of Medical Liability in Spain’ in Ewoud Hondius, *The Development of Medical Liability* (CUP 2010) 183.

59 See Bernard Berofsky, *Liberation from Self: A Theory of Personal Autonomy* (CUP 2007) 9.

60 Tom Beauchamp and James Childress, *Principles of Biomedical Ethics* (8th edn, OUP 2019) 72.

61 For the concept of Principlism, see ibid. 13-25.

62 In *Airdale NHS Trust v Bland* [1993] AC 789, 893, Lord Mustill notes that cases of mercy killing are illegal because ‘as in the other cases of consent to being killed, the interest of the state in preserving life overrides the otherwise all-powerful interest of patient autonomy’.

63 Beauchamp and Childress (n 60) 99.

64 Beauchamp and Childress (n 60) 102–3.

65 s 3, applicable to England and Wales only.

66 s 1(6), applicable to Scotland only.

67 *Pretty v UK* App no 2346/02 [2002] 35 EHRR 1, para 61.

68 Nor, indeed, are such instances limited to the common law world. In France, for instance, although ‘the Conseil d’Etat have to date refused to compensate specifically for loss of autonomy [*sine damno*]’, the courts in this jurisdiction have nevertheless afforded compensation in instances in which patients have suffered loss or injury as a result of physicians’ disregard for their rights of autonomy: Simon Taylor, ‘The Development of Medical Liability in France’ in Hondius (n 58) 104.

69 *Schloendorff v The Society of the New York Hospital* (1914) 211 NY 125, 105 NE 92, 95.

70 *Ciarlariello v Schacter* [1993] 2 SCR 119, 135.

71 [1993] Fam 95.

72 *Re T* (n 71) 113.

73 *Re T* (n 71) 113.

74 *Re T* (n 71) 115.

75 [2002] 2 All ER 449.

76 ibid 472.

77 *Airdale NHS Trust v Bland* (n 62) 864.

78 *Re C (Adult: Refusal of Medical Treatment)* [1994] 1 All ER 819.

79 The Age of Legal Capacity (Scotland) Act 1991, s 1(4) allows children under the age of 16 in Scotland to have capacity to consent to medical treatment, where a qualified medical practitioner is convinced that child is capable of understanding the nature and possible consequences of the proposed treatment, thereby echoing aspects of the guidance laid down following the English case of *Gillick v West Norfolk and Wisbech Area Health Authority* [1985] 3 All ER 402.

80 On this, see *Re J (A Minor) (Wardship: Medical Treatment)* [1990] 3 All ER 930.

81 [1990] Fam 26.

82 Above n 80.

83 *Re J* (n 80) 939.

84 *Wyatt v Portsmouth NHS Trust* [2005] EWCA Civ 1181, para 88.

85 *MB* (n 27) para 16.

86 This common law position stands in contrast to the position under Scots law, which—due to the operation of *parens patriae* and in line with the explanation of that jurisdiction discussed in *Gyngall* (n 22)—must explicitly substitute its judgement for those of the doctors or parents should a dispute ultimately lead to a petition to the jurisdiction of the Inner House.

87 ‘Instinctive’ relates to the innate human instinct to survive.

88 *MB* (n 27).

89 [2018] EWHC 2750 (Fam).

90 [1997] 1 WLR 242.

91 Above n 27. As is discussed below, this may not be the case in Scotland, however, if the *parens patriae* jurisdiction may only be exercised in cases concerning ‘infants who have had the misfortune to lose their parents’ in line with Lord Cranworth’s statement in *Stuart v Moore* (n 77) 910.

92 1993 Fam 15. This reluctance is also present in Scotland, as demonstrated in *Finlayson* (n 55), where parental refusal was overridden in favour of the clinicians who advocated the standard treatment. Meyers also refers to the strong tradition of deference to doctors’ clinical judgements in Scottish medical practice: David W Meyers, *‘*Letting Doctor and Patient Decide: The Wisdom of Scots Law’ in David W Meyers and David L Carey Miller (eds), *Comparative and Historical Essays in Scots Law: A Tribute to Professor Sir Thomas B Smith QC* (Butterworths 1992) 91

93 See *Law Hospital* (n 14) 329 (Lord Milligan).

94 *Law Hospital* (n 14) 319.

95 See Laurie, Harmon and Porter (n 9) para 4.21.

96 One of the few exceptions is Professor Laurie’s treatment of the jurisdiction: see Laurie (n 11) 95.

97 Laurie (n 11) 95; the King of Scots held this jurisdiction ‘since the days of Alexander II [1198–1249], and probably even of William the Lion [1142–1214]’, whereas in England ‘the history of the *parens patriae* jurisdiction begins in the reign of King Edward I [1272–1307]’: see Thomas Innes of Learney, *Scots Heraldry* (Oliver and Boyd 1956) 213; Paul LG Brereton, *The Origins and Evolution of the Parens Patriae Jurisdiction* (Lecture on Legal History, Sydney Law School, Friday 5 May 2017).

98 Further study of *parens patriae* in early Scots legal history would be welcome, but beyond the scope of this article; at present, Laurie’s article offers the most comprehensive treatment: see Laurie (n 11).

99 See Craig (n 13) 9.

100 Stair, *Institutes*, I, vi, 11.

101 See *Law Hospital* (n 14) 326 (Lord Cullen).

102 Thomson (n 13) 1.

103 See the comments of Lord Cringletie in *Bryce v Graham* (1828) 6 S 425.

104 Erskine, *Institutes*, I, vii, 9.

105 By virtue of s.1 of the Exchequer Court (Scotland) Act 1856.

106 See the discussion in Thomson (n 13) 120.

107 *Cumbria County Council v X* 2017 S.C. 451, para 25.

108 Thomson (n 13) 120.

109 (1861) 23 D 902.

110 See *Stuart v Moore* (n 109) 908 (the Lord Chancellor).

111 See *Stuart v Moore* (n 109) 908 (the Lord Chancellor).

112 Indeed, when the case was remitted to the Inner House, Lord Inglis said ‘it is truly lamentable, that the Court of Session, and the rules and principles which guide and regulate its proceedings, should be so little appreciated or understood’ in the English courts: see *Stuart v Moore* (n 109) 914 (the Lord Justice-Clerk).

113 *Stuart v Moore* (1860) 22 D 1504, 1507

114 The *nobile officium* has, indeed, been used in the 21st century as a means of recognising the otherwise unenforceable rulings of English courts: see *Cumbria CC, Petitioner* 2017 SC 451.

115 *Stuart v Moore* (1861) IX House of Lords Cases (Clark’s) 440, 441.

116 *Stuart v Moore* (n 109) 910 (Lord Cranworth).

117 *Stuart v Moore* (n 109).

118 *Stuart v Moore* (n 109).

119 *Stuart v Moore* (n 109).

120 *Stuart v Moore* (n 115) 469 (Lord Cranworth): ‘there is but one object which ought to be kept strictly in view, and that is, the interest of the infant’.

121 *Stuart v Moore* (n 109) 914 (the Lord Justice-Clerk).

122 *Stuart v Moore* (n 109) 914 (the Lord Justice-Clerk).

123 See the discussion in Thomson (n 13) 119; *Law Hospital* (n 14) 314 (Lord President Hope).

124 Mental Health Act 1959, s 150.

125 See *In re F (mental patient: sterilisation)* [1990] 2 AC 1, 58.

126 Laurie (n 11) 98.

127 See Laurie, Harmon and Porter (n 9) para 4.21.

128 *Law Hospital* (n 14).

129 *Law Hospital* (n 14) 324 (Lord President Hope).

130 Above n 62.

131 *Law Hospital* (n 14) 329 (Lord Milligan).

132 *Law Hospital* (n 14) 315 (Lord President Hope).

133 *Law Hospital* (n 14) 315.

134 *Law Hospital* (n 14) 315 (Lord President Hope).

135 *Law Hospital* (n 14) 319 (Lord President Hope).

136 *Law Hospital* (n 14) 316 (Lord President Hope).

137 *Law Hospital* (n 14) 318 (Lord President Hope).

138 *Law Hospital* (n 14) 319.

139 *Law Hospital* (n 14) 319.

140 *Law Hospital* (n 14) 305.

141 *Law Hospital* (n 14) 305.

142 See *Clarke v Fennoscandia Ltd* 2008 SC (HL) 122, para 27.

143 *Law Hospital* (n 14) 302.

144 *Law Hospital* (n 14) 305 (Lord President Hope).

145 *Law Hospital* (n 14) 329 (Lord Milligan).

146 Above n 62; see also Sheila AM McLean, ‘Permanent Vegetative State and the Law’ (2001) 71(s1) Journal of Neurology Neurosurgery and Psychiatry 26.

147 Though it was clear that the court found ‘the reasoning in that case wholly persuasive’: *Law Hospital* (n 14) 321 (Lord Clyde).

148 *Law Hospital* (n 14)316 (Lord President Hope).

149 *B (A Minor) (Wardship: Sterilisation), In re* [1988] AC 199.

150 *Law Hospital* (n 14) 316 (Lord President Hope).

151 As in *F* (n 125) and in *Bland* (n 62); see *Law Hospital* (n 14) 316 (Lord President Hope).

152 *Law Hospital* (n 14) 317 (Lord President Hope).

153 *Law Hospital* (n 14) 317.

154 *Irish Supreme Court, In the Matter of a Ward of Court* [1995] 2 ILRM 401.

155 *Law Hospital* (n 14) 315 (Lord President Hope).

156 *Law Hospital* (n 14) 321–4.

157 *Law Hospital* (n 14) 315 (Lord President Hope, concurring with Lord Clyde).

158 In contrast to the Court of Appeal and the UK Supreme Court (in non-Scottish cases), the Court of Session has no jurisdiction in criminal matters.

159 *Law Hospital* (n 14) 315 (Lord Clyde).

160 *Law Hospital* (n 14) 315.

161 1996 SCLR 516.

162 ibid.

163 See Laurie, Harmon and Porter (n 9) para 15.125.

164 Lord MacKay of Drumadoon, ‘Decision on the Persistent Vegetative State: Law Hospital’ (1996) paper presented at the Symposium on Medical Ethics and Legal Medicine, Royal College of Physicians and Surgeons of Glasgow (12 April 1996).

165 Laurie, Harmon and Porter (n 9) para 15.125.

166 See *Ross v Lord Advocate* [2016] CSIH 12, para 29 (Lord Carloway, then the Lord Justice-Clerk).

167 See the discussion in Laurie, Harmon and Porter (n 9) para 15.127.

168 *Ross* (n 166) para 29.

169 See the discussion in Laurie, Harmon and Porter (n 9) para 15.127.

170 Scottish Executive Policy Memorandum, 8 October 1999.

171 *Law Hospital* (n 14) 319 (Lord President Hope).

172 *Law Hospital* (n 14) 319.

173 See Laurie, Harmon and Porter (n 9) paras 15.121–15.127.

174 *Law Hospital* (n 14) 329 (Lord Milligan).

175 Laurie, Harmon and Porter (n 9) para 15.126.

176 In order to raise a private prosecution, the would-be prosecutor (if wishing to prosecute under solemn procedure, as would be necessary in a case of this kind) must apply, by bill, to the High Court for a grant of ‘criminal letters’. Such would not ordinarily be forthcoming without the support of the Lord Advocate, although such is not, strictly speaking, necessary. Nevertheless, there has only been one private criminal prosecution in Scotland within the last century: *X v Sweeny* 1982 JC 70. See Timothy H Jones and Ian Taggart, *Criminal Law* (7th edn, W Green 2018) para 2-53.

177 As in *Evans*, in which it was noted that the child’s parents could not comprehend the reality of the end-of-life care plan constructed by the clinicians: see *Evans* (n 36) paras 10–11.

178 That is, the fact that neither the parents of Charlie Gard nor Alfie Evans were married; the *patria potestas*, discussed below, operated only in respect of *legitimate* children at common law. The status of ‘illegitimacy’ has now been abolished, in Scotland, by s 21 of the Family Law (Scotland) Act 2006.

179 Norrie (n 9) para 1.28.

180 David W Meyers, *The Human Body and the Law* (2nd edn, Stanford UP 1990) 156.

181 ibid.

182 Again; provided that the children were ‘legitimate’.

183 *Patria potestas* was, in spite of its nigh unlimited nature, not a power comparable to *dominium* or ‘ownership’ in Roman or Scots law, even notwithstanding the fact that infant and pupil children are conceptualised as ‘property’ for the purposes of Scots criminal law: see Jonathan Brown, ‘Plagium: An Archaic and Anomalous Crime’ [2016] Jur Rev 129, *passim*.

184 Norrie (n 9) para 1.28.

185 Recall Sir Thomas Craig (n 13).

186 From s 10 of that Act.

187 See Kenneth McK Norrie, ‘Legislative Background to the Treatment of Children and Young People Living Apart from their Parents: Report for the Scottish Child Abuse Inquiry (2018) 14.

188 Norrie (n 9) para 1.04.

189 Norrie (n 9) para 7.34.

190 Norrie (n 9) para 1.28.

191 To paraphrase Meyers (n 92) 97.

192 See the discussion in Meyers (n 92) 91–3.

193 Lord President Clyde famously attributed the comparable absence of Scottish claims of physician malpractice to ‘the high standard in general of the medical profession in Scotland’: see *Hunter v Hanley* 1955 SC 200, 205. The judiciary seems to have consistently endorsed this assessment: see *Moyes v Lothian Health Board* 1990 SLT 444.

194 See the discussion in Graeme Laurie, ‘Personality, Privacy and Autonomy in Medical Law’ in Niall R Whitty and Reinhard Zimmermann (eds), *Rights of Personality in Scots Law: A Comparative Perspective* (Dundee UP 2009) 454–7.

195 *Montgomery v Lanarkshire Health Board Scotland* [2015] UKSC 11, para 75.

196 Gillett (n 6).

197 Meyers (n 92) 97.

198 See eg *HM Advocate v Ross* National Archives of Scotland, JC26/1967/117, High Court of Edinburgh trial papers, 24 January 1967.

199 See Meyers (n 92) 92.

## Notes

The authors thank the referees, and their colleague Dr Craig Anderson, for their constructive and valuable comments.

